# Is Crocin Effective in Modulating Blood Lipid Levels? An Updated Systematic Review and Meta-Analysis with Dose– and Time–Response Assessments

**DOI:** 10.3390/ph18111735

**Published:** 2025-11-14

**Authors:** Lucas Fornari Laurindo, Eduardo Federighi Baisi Chagas, Victória Dogani Rodrigues, Ricardo de Argollo Haber, Flávia Cristina Castilho Caracio, Maria Clara Capobianco Marangão, Manuela dos Santos Bueno, Eliana de Souza Bastos Mazuqueli Pereira, Cláudia Rucco Penteado Detregiachi, Vitor Engrácia Valenti, Mayara Longui Cabrini, Sandra Maria Barbalho

**Affiliations:** 1 Graduate Program in Medical Sciences, Division of Cellular Growth, Hemodynamic, and Homeostasis Disorders, Faculdade de Medicina, Universidade de São Paulo (USP), São Paulo 05403-000, SP, Brazil; 2 Laboratory for Systematic Investigations of Diseases, Department of Biochemistry and Pharmacology, School of Medicine, Universidade de Marília (UNIMAR), Marília 17525-902, SP, Brazil; 3 Postgraduate Program in Structural and Functional Interactions in Rehabilitation, School of Medicine, Universidade de Marília (UNIMAR), Marília 17525-902, SP, Brazil; 4 Department of Biochemistry and Pharmacology, School of Medicine, Faculdade de Medicina de Marília (FAMEMA), Marília 17519-030, SP, Brazil; 5 Department of Medical Sciences and Education, School of Medicine, Universidade de Marília (UNIMAR), Marília 17525-902, SP, Brazil; 6 Laboratory for the Evaluation of Pharmacological and Non-Pharmacological Interventions on Cardiometabolic Health, Faculty of Philosophy and Sciences, Universidade Estadual Paulista (UNESP), Marília Campus, Marília 17525-900, SP, Brazil; 7 Department of Physiotherapy, School of Physiotherapy, Universidade de Marília (UNIMAR), Marília 17525-902, SP, Brazil; 8 Department of Biochemistry and Nutrition, School of Food and Technology of Marília (FATEC), Marília 17500-000, SP, Brazil; 9 Department of Research, Research Coordination Center, UNIMAR Charitable Hospital, Universidade de Marília (UNIMAR), Marília 17525-902, SP, Brazil

**Keywords:** crocin, carotenoid, lipids, LDL-C, HDL-C, triglycerides, total cholesterol, dyslipidemia

## Abstract

**Background/Objectives:** Dyslipidemia is a global health concern. It refers to increased blood levels of LDL-C, triglycerides, and total cholesterol, accompanied by decreased blood HDL-C levels. Many pharmacological and non-pharmacological approaches have been designed to improve dyslipidemia management. However, nutritional therapies have gained more attention due to their antioxidant and anti-inflammatory properties. In this scenario, the carotenoid crocin stands out as a prominent anti-dyslipidemia phytochemical. Its unique structure permits lipid-lowering effects via various mechanisms, including the enhancement of lipid breakdown, reduction in lipid formation, bolstering of antioxidant defenses to diminish lipid toxicity, and decreased absorption of dietary fats. However, no recent systematic review or meta-analysis has addressed its anti-dyslipidemia effects with statistical power. Therefore, we aim to fill this gap with our current meta-analysis, as well as dose and time–response assessments. **Methods:** PubMed, SpringerLink, ScienceDirect, Cochrane, and Google Scholar databases were searched, and PRISMA guidelines were followed. Ten studies comprising eleven results were included. **Results:** Crocin did not improve LDL-C (0.2120, 95% CI: −0.0799 to 0.5040), HDL-C (−0.1937, 95% CI: −0.4896 to 0.1022), triglyceride (−0.2063, 95% CI: −0.5764 to 0.1638), or total cholesterol (0.1528, 95% CI: −0.1074 to 0.4129). The dose–response or time–response was also not statistically significant. **Conclusions:** More clinical studies with robust designs must be conducted to thoroughly assess crocin’s effectiveness in modulating lipid levels with the utmost care.

## 1. Introduction

Despite the high reported rates, dyslipidemia is likely underdiagnosed. It is often asymptomatic unless accompanied by other cardiometabolic diseases, making it a “silent” condition. People aged 20–45 years are screened at low rates (<50%), and approximately half of the individuals between 20 and 39 years old meet the guidelines for lipid issues [1]. Dyslipidemia refers to abnormal levels of blood lipids [2]. Notably, key components in these processes are high-density lipoprotein cholesterol (HDL-C) levels, alongside low-density lipoprotein cholesterol (LDL-C), triglyceride, and total cholesterol levels [3]. These abnormalities significantly increase the risk of cardiovascular diseases and outcomes, including coronary artery disease and atherosclerosis. Conventional therapies for dyslipidemia include statins (HMG-CoA reductase inhibitors), PCSK9 inhibitors, cholesterol absorption inhibitors, bile acid sequestrants, and, more recently, nutritional supplements [4,5].

**Figure 1:**
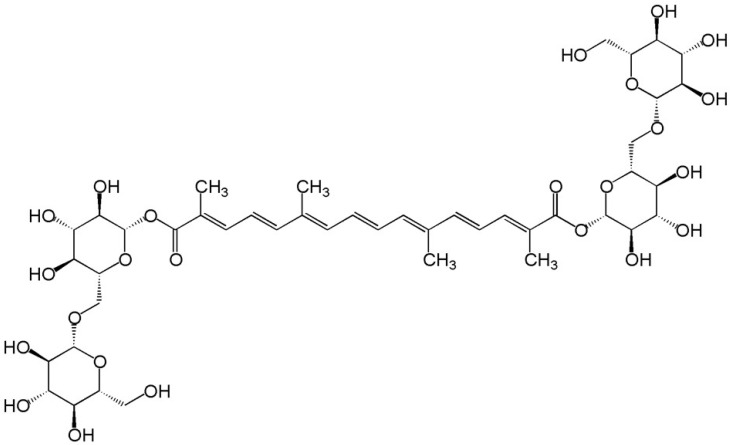
Molecular Structure of Crocin.

**Figure 2:**
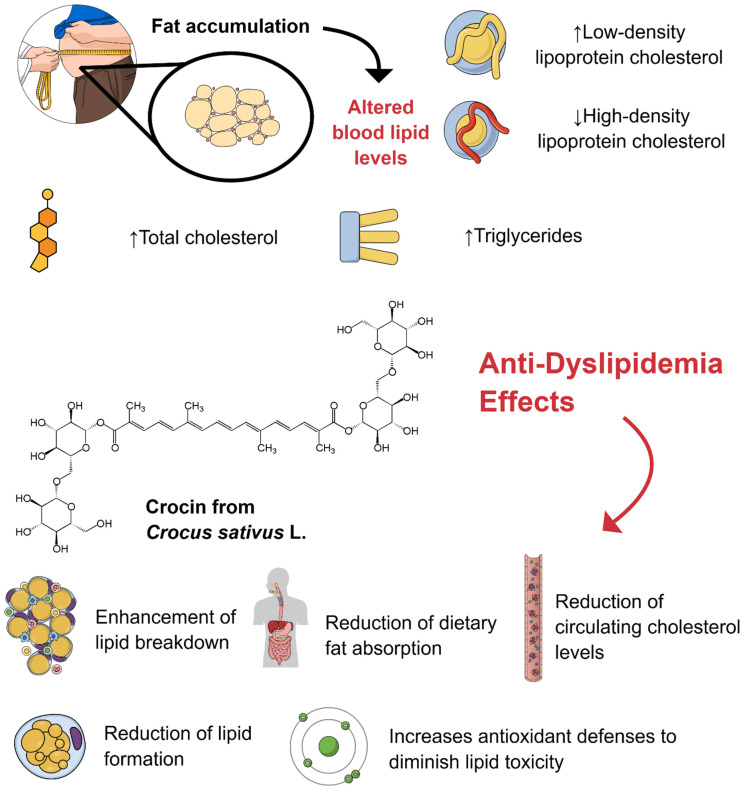
Anti-Dyslipidemia Effects of Crocin. Dyslipidemia refers to abnormal levels of lipids (fats) in the blood, which may include elevated levels of low-density lipoprotein cholesterol, total cholesterol, or triglycerides, and/or low levels of high-density lipoprotein cholesterol. Crocin, a carotenoid compound, has been extensively studied for its lipid-lowering and antioxidant properties. This compound works through a combination of complementary mechanisms to regulate lipid metabolism and mitigate dyslipidemia. It exerts beneficial effects through several mechanisms, including enhanced lipid breakdown, reduced dietary fat absorption, lowered circulating cholesterol levels, reduced lipid formation, and reduced lipid lipotoxicity due to its antioxidant activity, ultimately helping to manage dyslipidemia. The first part of the figure refers to the clinical and laboratory settings for dyslipidemia diagnosis. The described mechanisms of action were based on the studies of Ali et al., Demir et al., Bastani et al., and Yaribeygi et al. [17,18,19,21]. ↑: increase; ↓: decrease. Created using Mind the Graph (https://mindthegraph.com/).

Medical nutritional therapies have been widely discussed for improving metabolic and lipid profiles [6]. They are tailored to the type of dyslipidemia and associated cardiovascular risk factors. In this scenario, supplements containing monounsaturated fatty acids (MUFAs), polyphenols, phytosterols, and carotenoids are crucial, as they support cardiovascular function, vascular health, and metabolic health [7]. Crocin (C_44_H_64_O_24_) is a water-soluble carotenoid predominantly found in saffron (*Crocus sativus* L.) and gardenia [8]. This compound has garnered significant attention in scientific research due to its extensive array of beneficial properties, which include antioxidant [9], anti-inflammatory [10], neuroprotective [11], cardioprotective [12], anticancer [13], antiatherosclerosis [14], antidepressant [15], and nephroprotective [16] effects. Crocin is responsible for the vibrant red and golden-yellow hues associated with saffron. From a chemical perspective, it is characterized as a diglycosyl or monoglycosyl ester of crocetin, synthesized through esterification with gentiobiose, comprising two β-linked D-glucose units. Its capacity to combat dyslipidemia has been demonstrated through several mechanisms, including the enhancement of lipid breakdown, reduction in lipid formation, bolstering of antioxidant defenses to mitigate lipid toxicity, and decreased absorption of dietary fats along with a reduction in circulating cholesterol levels [17,18]. Figure 1 illustrates the chemical composition of crocin, highlighting its molecular structure. Figure 2 illustrates the main pathways by which crocin modulates blood lipid levels.

Preclinical data indicated that crocin inhibits lipogenesis by suppressing the expression of key regulatory proteins involved in lipogenesis and upregulating proteins in lipid catabolism [19]. Crocin reduces the expression of key genes related to lipogenesis, including the sterol regulatory element binding protein-1c (SREBP-1c), fatty acid synthase (FAS), stearoyl-CoA desaturase 1 (SCD1), peroxisome proliferator-activated receptor-γ (PPAR-γ), diacylglycerol acyltransferase (DGAT), and CCAAT/enhancer binding protein α (CEBPα). Additionally, crocin upregulates the genetic expression of peroxisome proliferator-activated receptor-α (PPARα), lipoprotein lipase, and hormone-sensitive lipase. Therefore, it reduces adipogenesis and promotes adipolysis while reducing lipid accumulation [20]. Crocin also upregulates the genes for superoxide dismutase (SOD), catalase (CAT), and glutathione peroxidase (GPx), thereby enhancing the antioxidant defense system, reducing free radical generation, and mitigating lipotoxicity [21].

Previously, Naserizadeh et al. [20] conducted a systematic review and meta-analysis of randomized controlled trials to assess the effectiveness of crocin supplementation in improving blood lipid levels. Their results found that crocin supplementation could significantly decrease fasting blood glucose levels and total cholesterol. However, they did not find beneficial effects on triglyceride, LDL-C, and HDL-C levels. Although this previous manuscript has its merits, its analysis lacked specificity, as it evaluated blood glucose levels in conjunction with lipids. Additionally, since the publication of this manuscript, numerous new results have been reported. Therefore, there is a need to update the previous analysis with an updated version of the results. Our current systematic review and meta-analysis aim to fill this gap in the literature by conducting a comprehensive literature review following the Preferred Reporting Items for Systematic Reviews and Meta-Analyses (PRISMA) guidelines. Our search strategy included studies comprising eleven results from reputable databases, such as PubMed. To improve our results, our systematic review and meta-analysis were also strengthened with a dose– and time–response assessment.

Our study fills a significant gap in current literature. It provides a comprehensive meta-analysis of the effects of crocin supplementation on human lipid profiles, based on the totality of available publications, including those published recently. While previous reviews have compiled available evidence, none have included recent trials or examined the dose–response relationship and time-dependent effects of crocin. Additionally, most existing research has focused on preclinical data. Our study is the first to combine clinical findings with mechanistic insights, offering a more complete understanding of crocin’s potential as a therapeutic agent for dyslipidemia.

## 2. Materials and Methods

### 2.1. Focused Question

This systematic review and meta-analysis were designed to answer the question, “*What are the effects of Crocin on improving the lipid profile of human subjects?*”

### 2.2. Language

This review exclusively considered trials that were initially published in the English language. While this may be perceived as a potential bias, a prior review article has indicated that there is no evidence of a systematic bias in literature reviews resulting from restrictions related to the English language [22].

### 2.3. Literature Search and Databases

We conducted a literature review by searching the PubMed, SpringerLink, ScienceDirect, Cochrane, and Google Scholar databases. We utilized the mesh terms “Crocin,” “HDL-C,” “LDL-C,” “low-density lipoprotein cholesterol,” “high-density lipoprotein cholesterol,” “lipids,” “blood lipids,” “cholesterol,” “triglycerides,” “Metabolism,” and “total cholesterol.” We also utilized Boolean operators, such as “AND” or “OR”, to refine the literature search process. All acknowledged articles were imported into the Rayyan QCRI (Qatar Computing Research Institute, Qatar, https://www.hbku.edu.qa/en/qcri, accessed 29 August 2025) to facilitate the elimination of duplicates. The software conducted an initial screening of the studies by analyzing their titles and abstracts. L.F.L. and V.E.V. undertook the suitability assessment by reviewing the selected manuscripts for post-duplication screening. In cases of disagreement regarding the inclusion of specific studies, an additional reviewer (S.M.B.) made the final determination. The authors subsequently evaluated the feasibility of conducting a meta-analysis of the included studies that met the established eligibility criteria. It is important to note that this systematic review and meta-analysis do not possess a registration number.

### 2.4. Study Selection

All studies included in this review were sourced from esteemed peer-reviewed journals. These publications span the database’s inception through September 2025. The PICOS framework (Population, Intervention, Comparison, Outcomes, and Study Design) served as the basis for establishing the inclusion and exclusion criteria.

(P) Patients were older than 18 years old.

(I) The intervention group ought to receive crocin as an intervention. All treatment durations and doses were accepted throughout our literature selection process.

(C) We only included studies that evaluated human subjects within clinical trials. The human participants should have received a placebo to control for the effect of comparisons between groups.

(O) The levels of LDL-C, HDL-C, triglycerides, and total cholesterol were the primary outcomes of interest for this systematic review and meta-analysis.

(S) All included studies were either randomized and placebo-controlled or had a control group. We only considered articles published in reputable, peer-reviewed journals and written in the English language. We excluded conference papers, abstracts, master’s dissertations, doctoral theses, descriptive studies, commentaries, case studies, editorials, and reviews. However, we referenced previous reviews on the general effects of crocin to inform some sections of our current manuscript, including the Introduction. The selected studies have a time range of up to 2025. It means that all available studies, regardless of the publication date, were considered for inclusion. Our search was not restricted to publication date, and we aimed to include all studies that met the pre-specified inclusion criteria. This search was conducted through September 2025, ensuring that the most recent research was also included in the final analysis.

### 2.5. Data Extraction

We collected data on the author, year of publication, study design, number of participants, demographic information, and details about the intervention protocols for each study included. This information was extracted from primary studies and organized in a table. Any significant missing data were requested by reaching out to the corresponding authors of the studies. Two reviewers, E.F.B.C. and V.D.R., completed this stage independently of each other. When the corresponding authors did not respond, we used Web Plot Digitizer^®^ to extract data from published graphs in the primary articles. Data regarding lipid profiles were recorded as the mean and standard deviation. Values presented as “standard error” or “confidence intervals” in the primary studies were converted to standard deviation. When only the median and interquartile range were available, the mean and standard deviation were estimated using the method proposed by Wan et al. [23].

### 2.6. Search and Selection of Relevant Articles

We collected data for this manuscript in accordance with the PRISMA guidelines, as reported by Page et al. The PRISMA checklist has also been used for writing the main text and abstract [24].

### 2.7. Data Items

We collected the means and standard deviations for LDL-C, HDL-C, triglycerides, and total cholesterol values. Additionally, we obtained data regarding participant and intervention profiles, funding sources, and support mechanisms from the selected references included in the analysis. At this stage, any missing or ambiguous information has been excluded from consideration.

### 2.8. Quality Assessment

The Cochrane Handbook for Intervention Assessments [25] was used to evaluate the bias of the included studies. Information regarding question focus, appropriate randomization, allocation blinding, participant losses, prognostic and demographic characteristics, outcomes, intention-to-treat analysis, sample calculations, and adequate time for follow-up was gathered from the included studies and presented within a table.

### 2.9. Qualitative Analysis

A narrative synthesis was conducted to provide a comprehensive account of the completion details for each study. The specifics of each study were presented through a combination of textual descriptions and tabular formats. The outcomes relating to lipid intervention through crocin supplementation were derived by comparing the results from the intervention group with those from the control (placebo) group.

### 2.10. Synthesis of Results and Summary Measures

The statistical analysis was conducted utilizing the Jamovi open statistics software (Version 2.6.26, Solid). A *p*-value of less than 0.05 was established as the threshold for determining statistical significance. In executing this meta-analysis, the standardized mean difference was employed as the outcome measure, and a random-effects model was applied to the dataset [25]. Heterogeneity, denoted as tau^2^, was estimated utilizing the DerSimonian–Laird estimator. Furthermore, we also reported the Q-test for heterogeneity along with the I^2^ statistic. In cases where heterogeneity was identified (i.e., tau^2^ > 0, independent of the Q-test results), we predicted the interval for the true outcomes. To explore the impact of exposure, dose– and time–response assessments were examined. We employed studentized residuals and Cook’s distances to assess the potential for outliers and influential observations within the model. A study was deemed a potential outlier if its studentized residual exceeded the 100 × (1 − 0.05/(2 × k))^th^ percentile, where k represents the number of results included in the analysis. This determination was based on the assumption of deviation from a standard normal distribution, specifically utilizing the Bonferroni correction test with a two-sided α = 0.05 for the results incorporated in this meta-analysis. Moreover, studies were deemed influential if they exhibited a Cook’s distance exceeding the median plus six times the interquartile range of the Cook’s distances. To assess funnel plot asymmetry, both the rank correlation test and the regression test were employed, utilizing the standard error of the observed outcomes as a predictive variable [26,27,28,29,30,31,32,33,34].

## 3. Results and Discussion

### 3.1. Literature Search Report

**Figure 3:**
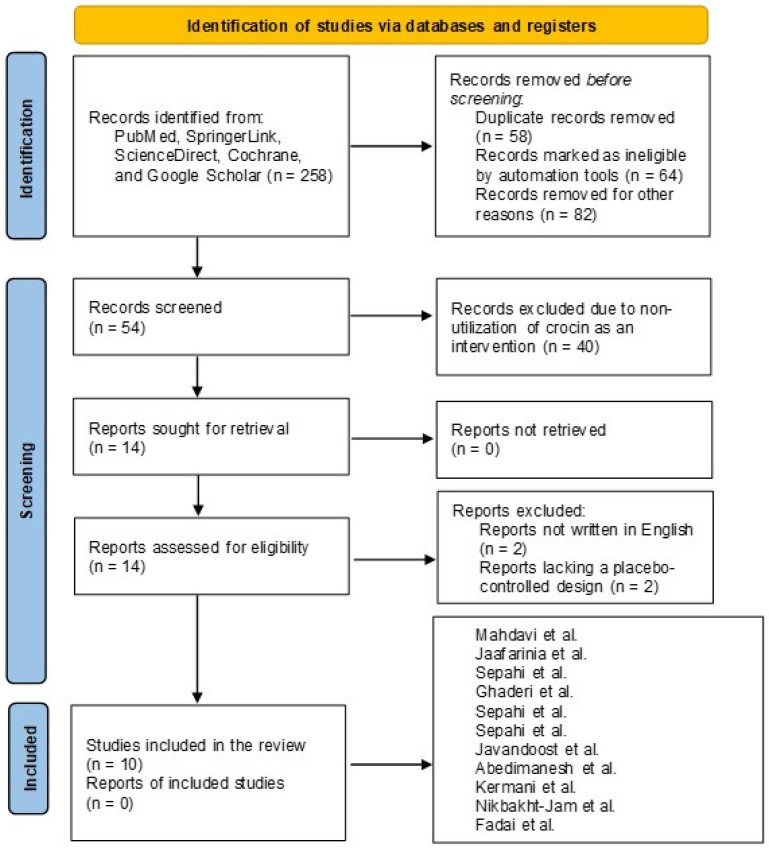
Illustration of the Literature Search Process following the PRISMA guidelines [35,36,37,38,39,40,41,42,43,44].

Figure 3 depicts the literature search process following the guidelines reported by the PRISMA group. Following initial screening, 258 reports were identified from databases, including PubMed, SpringerLink, ScienceDirect, Cochrane, and Google Scholar. At this stage, 58 reports were excluded due to duplication, 64 were marked as ineligible by automation tools, and 82 were removed for other reasons, including inconsistencies in methodology between our inclusion and exclusion criteria. Fifty-four records remained for screening, and 40 were excluded because they did not utilize crocin as an intervention. At this stage, 14 reports were sought for retrieval, and all reports were successfully retrieved. Following retrieval, two reports were excluded because they were not written in English, and two additional reports were excluded due to a lack of a placebo-controlled design. Finally, ten reports remained and were included in the final analysis. Because one study utilized data on different dosages of crocin, all reports were described in the PRISMA flowchart.

### 3.2. Overview of the Included Studies and Bias Assessment

Mahdavi et al. [35] conducted a randomized, double-blind, placebo-controlled study to evaluate the effects of crocin supplementation (30 mg/d) in the form of tablets in a sample containing 23 smokers (34.39 ± 10.72 years) for three months. The results indicated null effects regarding crocin supplementation against lipid profile parameters, including triglycerides (173.77 ± 19.20 mg/dL → 172.61 ± 17.44 mg/dL, *p* = 0.84), total cholesterol (198.38 ± 22.29 mg/dL → 197.23 ± 21.64 mg/dL, *p* = 0.82), LDL-C (123.60 ± 21.47 mg/dL → 123.08 ± 20.40 mg/dL, *p* = 0.92), and HDL-C (40.02 ± 5.32 mg/dL → 39.62 ± 5.45 mg/dL, *p* = 0.81), compared to placebo. However, the small sample size and the limited intervention duration might be limiting factors influencing the results. Although the crocin intervention did not yield significant results regarding lipid profile parameters, it promoted statistically significant improvements in fasting plasma glucose, insulin, and insulin resistance levels, indicating positive effects of crocin that led to improved metabolic regulation regarding blood glucose levels. Crocin also presented statistically significant improvements in pro-inflammatory parameters, including substantial reductions in high-sensitivity C-reactive protein (hs-CRP) levels. Future research endeavors should focus on conducting larger, adequately powered clinical trials with larger sample sizes and treatment durations to overcome the statistical limitations of small cohorts, thereby enabling reliable detections of potential lipid-modulating effects of crocin in smokers.

In another study, Jaafarinia et al. [36] employed a triple-blind, randomized, placebo-controlled approach to evaluate the effects of crocin (15 mg/d for 90 days) on the metabolic parameters of twenty-one patients (63.86 ± 10.62 years, 27.21 ± 3.86 kg/m^2^) diagnosed with diabetic nephropathy. Crocin did not significantly alter total cholesterol (133.24 ± 29.49 mg/dL → 141.81 ± 40.97 mg/dL, *p* = 0.26), HDL-C (41.62 ± 9.93 mg/dL → 45.95 ± 12.28 mg/dL, *p* = 0.84), and LDL-C (64.51 ± 23.44 mg/dL → 63.95 ± 33.57 mg/dL, *p* = 0.43) levels, compared to placebo. However, crocin intervention significantly improved triglyceride levels (157.89 ± 84.69 mg/dL → 133.79 ± 43.21 mg/dL, *p* = 0.03). The intervention lasted for only a limited period, and the dose may not be sufficient to achieve better results. In the present study, the levels of pro-inflammatory cytokines, including transforming growth factor-β (TGF-β), decreased following crocin intervention. Still, the results did not yield statistical significance. Since triglyceride levels were reduced in diabetic nephropathy patients, future clinical studies should assess crocin’s efficacy in a broader range of participants, including those with comorbidities such as diabetes, chronic kidney disease, or hypertriglyceridemia, diseases in which triglyceride control is particularly relevant for cardiovascular outcomes. Additionally, given that the studied population had diabetic nephropathy, future studies must explore whether triglyceride modulation is related to improved kidney function or reductions in renal inflammation parameters and fibrosis (through, for example, the modulation of TGF-β signaling pathways).

In a randomized, triple-blind, placebo-controlled trial, Sepahi et al. [37] evaluated the effects of crocin (30 mg/d for three months) supplementation on diabetic patients (57.58 ± 1.0 years). They found that crocin may be effective in counteracting fasting blood glucose and glycated hemoglobin levels in patients with type 2 diabetes mellitus. However, the intervention did not yield significant improvements regarding total cholesterol (184.47 ± 40.28 mg/dL → 187.55 ± 42.84 mg/dL), triglycerides (174.84 ± 7.49 mg/dL → 174.75 ± 4.60 mg/dL), HDL-C (47.46 ± 10.21 mg/dL → 46.22 ± 2.14 mg/dL), and LDL-C (102.86 ± 39.05 mg/dL → 105.08 ± 41.72 mg/dL) levels. Despite not reporting severe limitations, future research endeavors emerge from this specific study. Since crocin improved glycemic control markers but did not alter lipid levels, future research trials should aim to investigate whether the glycemic benefits indirectly contribute to long-term improvements in lipid metabolism when observed over more extended intervention periods. Higher doses of crocin may also be used in this regard to observe if the benefits regarding lipid modulation with crocin in diabetic patients are dose-dependent. Using subgroup analysis within diabetic and hyperlipidemic populations, such as those with diabetes and isolated hypertriglyceridemia, could also significantly enhance the understanding of how crocin modulates lipid parameters.

Ghaderi et al. [38] conducted a double-blind, randomized, placebo-controlled clinical study to assess the effects of a crocin supplement (30 mg/d for eight weeks) on the health statuses of Iranian patients undergoing methadone maintenance treatment (*n* = 26, 44.5 ± 9.4 years, 24.5 ± 4.4 kg/m^2^). Regarding lipid regulation, crocin yielded significant results in decreasing total cholesterol levels (196.8 ± 39.0 mg/dL → 191.4 ± 37.8 mg/dL, *p* = 0.03) and triglyceride levels (169.0 ± 57.1 mg/dL → 148.8 ± 44.2 mg/dL, *p* = 0.001) compared to placebo. However, the results were not significant for HDL-C levels (45.5 ± 6.4 mg/dL → 46.6 ± 6.5 mg/dL, *p* = 0.24) and LDL-C levels (117.5 ± 36.0 mg/dL → 115.1 ± 33.7 mg/dL, *p* = 0.49) compared to placebo. The placebo and intervention groups were similar in terms of baseline characteristics. Therefore, the reliability of the results might have been strengthened. However, due to the small sample size, replicating this treatment strategy in larger-scale studies might be essential to confirm the lipid-lowering effects of crocin in patients undergoing psychiatric treatments. Studies must also elucidate more profoundly whether crocin counteracts methadone-associated metabolic disruptions through antioxidant, anti-inflammatory, or hepatic regulation pathways, including insulin resistance, inflammatory biomarkers, oxidative stress indices, and liver function markers as secondary outcomes.

In another study, Sepahi et al. [39] conducted a randomized, double-blind, placebo-controlled, phase 2 clinical study to evaluate the effects of crocin supplementation in patients with diabetic maculopathy. Patients received 5 mg or 15 mg/d of crocin tablets daily for three consecutive months. The effects were not significant regarding lipid profile parameters, including LDL-C (118.4 ± 3.46 mg/dL → 115.38 ± 5.9 mg/dL in the 15 mg/d group and 123.7 ± 8.63 mg/dL → 121.85 ± 7.93 mg/dL in the 5 mg/d group), HDL-C (44.25 ± 1.41 mg/dL → 42.7 ± 1.19 mg/dL in the 15 mg/d group and 42.85 ± 1.79 mg/dL → 43.2 ± 1.61 mg/dL in the 5 mg/d group), triglycerides (200.95 ± 19.84 mg/dL → 201.6 ± 17.43 mg/dL in the 15 mg/d group and 198.3 ± 13.65 mg/dL → 190.8 ± 11.47 mg/dL in the 5 mg/d group), and total cholesterol (199.3 ± 7.7 mg/dL → 198.65 ± 7.14 mg/dL in the 15 mg/d group and 193.65 ± 9.01 mg/dL → 199.4 ± 7.89 mg/dL in the 5 mg/d group). Limited sample size is the major limitation of this study, as mentioned by the authors. Additionally, since 5 mg and 15 mg/d were ineffective for patients with severe diabetic complications, including diabetic maculopathy, future research endeavors must aim to test whether higher doses produce better lipid-modulating effects in this population. In this sense, conducting randomized multi-center studies might be essential to validate the findings. Future studies should also assess whether crocin’s metabolic effects, including lipid, glycemic, and inflammatory modulation, correlate with improvements in retinal health, visual acuity, or disease progression in diabetic maculopathy.

Kermani et al. [40] conducted a randomized, double-blind, placebo-controlled clinical study to evaluate the effects of crocin supplementation (100 mg/d) on metabolic syndrome parameters over a 6-week period. Patients with metabolic syndrome, as defined by the International Diabetes Federation (IDF), were enrolled, and the crocin dosage was well-tolerated. After the experimental protocol finished, the researchers indicated positive outcomes regarding crocin modulation of total cholesterol (230.1 ± 42.3 mg/dL → 204.5 ± 41.2 mg/dL, *p* < 0.001) and triglyceride (218.1 ± 80.0 mg/dL → 173.8 ± 97.5 mg/dL, *p* = 0.003) levels, with statistically significant improvements. Unfortunately, HDL-C (40.3 ± 8.4 mg/dL → 40.0 ± 7.8 mg/dL, *p* = 0.85) and LDL-C (146.3 ± 25.4 mg/dL → 139.1 ± 25.8 mg/dL, *p* = 0.48) levels have not shown significant improvement after 6 weeks of crocin intervention. However, the study presents limitations, such as the fact that dietary intake and physical activity were not adjusted during the study intervention, which are confounding factors that may have impacted the results. Additionally, the sample was considered low. Since higher doses of crocin (100 mg/d) produced significant lipid improvements, future studies should aim to validate higher doses of this bioactive compound against lipid parameters, to establish the minimal effective dose and potential therapeutic thresholds for potent lipid modulation. Additionally, future research should aim to adjust cofounding factors, such as diet, caloric intake, and physical activity, to isolate crocin’s independent effects on lipid metabolism and to determine whether lifestyle modifications potentiate crocin’s effects. Finally, since metabolic syndrome is a significant health concern, comparative efficacy with standard crocin therapies must be conducted in larger, stratified metabolic syndrome cohorts across different dominant components, such as obesity-dominant vs. insulin resistance-dominant phenotypes.

Javandoost et al. [41] conducted a randomized, double-blind, placebo-controlled clinical trial to evaluate the effects of crocin supplementation 30 mg/d in twenty-one patients with metabolic syndrome. The primary aim was to observe the effects of crocin on the lipid profile. At the final of the study protocol, no statistically significant changes were observed regarding total cholesterol (232.18 ± 66.52 mg/dL → 220.09 ± 55.60 mg/dL, *p* = 0.702), triglyceride (151.00 (111.50–204.25) mg/dL → 160.50 (102.00–227.25) mg/dL, *p* = 0.080), LDL-C (163.50 (120.25–204.25) mg/dL → 121.00 (102.00–170.75) mg/dL, *p* = 0.986), and HDL-C (37.00 (31.75–46.00) mg/dL → 50.00 (40.50–56.25) mg/dL, *p* = 0.687) modulation, compared to placebo. Fasting blood glucose levels have also not been significantly improved by crocin supplementation (*p* = 0.614). Limitations include a small sample size, resulting in low statistical power for the analyses. In this sense, larger, adequately powered trials are needed to overcome type II error and to assess whether crocin exerts modest but significant effects on lipid profile parameters in patients with metabolic syndrome. In terms of metabolic syndrome, trials must also stratify patients by baseline characteristics, including dyslipidemia, glycemic control, and obesity severity, as crocin may affect these distinct components of the syndrome differently in terms of phenotype severity.

In an eight-week randomized, double-blind, placebo-controlled clinical study conducted on patients with coronary artery disease, Abedimanesh et al. [42] evaluated the effects of daily supplementation with 30 mg crocin for eight weeks vs. placebo on appetite, dietary intakes, and body composition. Crocin supplementation did not significantly improve lipid profile parameters. Therefore, lipid profile parameters, including triglyceride (182.37 ± 87.27 mg/dL → 192.32 ± 101.00 mg/dL), total cholesterol (166.26 ± 32.66 mg/dL → 172.11 ± 33.87 mg/dL), HDL-C (45.84 ± 6.52 mg/dL → 47.84 ± 8.33 mg/dL), and LDL-C (81.31 ± 28.47 mg/dL → 83.21 ± 26.23 mg/dL) have not been significantly modulated. However, the small sample size and the short duration of treatment may be limitations that influenced the lack of positive results. Since coronary artery disease patients possess a higher risk for dyslipidemia progression, studies with extended intervention durations are necessary to determine whether crocin supplement exerts delayed effects on lipid regulation in this population. Additionally, since coronary artery disease is highly associated with oxidized lipoproteins, future studies should prioritize analyzing the effects of crocin supplementation on oxidized LDL-C particles, as well as markers of endothelial function, to fully capture subtler benefits of crocin in individuals with coronary artery disease.

Nikbakht-Jam et al. [43] conducted an eight-week randomized, placebo-controlled, double-blind clinical trial with patients having metabolic syndrome to evaluate whether 30 mg/d of crocin supplementation could affect the antioxidant statuses of the patients (intervention group: *n* = 29, 38.97 ± 13.33 years). At the end of the experiment, no statistically significant changes (*p* > 0.05) were observed regarding lipid profile parameters, including LDL-C (152.29 ± 56.93 mg/dL → 123.52 ± 48.06 mg/dL), HDL-C (38.59 ± 10.14 mg/dL → 49.25 ± 11.05 mg/dL), total cholesterol (224.48 ± 60.83 mg/dL → 210.52 ± 52.68 mg/dL), and triglyceride levels (147.50 (113–199) mg/dL → 132.00 (108–201) mg/dL) compared to placebo. No positive outcomes were associated with fasting blood glucose modulation as well (*p* > 0.05). A significant limitation of this study is that serial assessments were not conducted during the intervention protocol. Therefore, the assessments were conducted only at the beginning and at the end of the experiment period. In this sense, future trials should incorporate multiple time-point evaluations during the intervention period to capture the dynamic changes that crocin supplementation may exert on lipid parameters, including those that occur both acutely and chronically following the treatment duration. Longitudinal designs must be introduced to clarify whether crocin’s effects emerge early, plateau, or require sustained intake over time.

Using a randomized, triple-blind, placebo-controlled design, Fadai et al. [44] evaluated the effects of crocin (*n* = 20, 48.1 ± 7.7 years, administered at 30 mg daily for two, six, and twelve weeks) on patients diagnosed with schizophrenia who were undergoing olanzapine treatment. The treatment strategy did not significantly modulate plasma lipid levels, including triglyceride (101.0 ± 55.6 mg/dL → 103.7 ± 47.9 mg/dL → 125.1 ± 59.5 mg/dL → 102.8 ± 47.1 mg/dL), total cholesterol (181.5 ± 34.2 mg/dL → 172.4 ± 32.0 mg/dL → 182.2 ± 32.5 mg/dL → 178.9 ± 34.4 mg/dL), LDL-C (111.2 ± 32.4 mg/dL → 106.7 ± 25.9 mg/dL → 114.8 ± 30.3 mg/dL → 112.5 ± 28.3 mg/dL), and HDL-C (45.6 ± 9.7 mg/dL → 46.4 ± 11.9 mg/dL → 48.1 ± 11.9 mg/dL → 51.3 ± 9.7 mg/dL) levels. The groups were similar in terms of baseline characteristics, including age and olanzapine daily dose, as well as comorbidity duration, which reinforces the validity of the result analyses.

Table 1 reports the characteristics of the included studies following the PRISMA Guidelines for Reporting Interventions [24]. Table 2 presents the biases in the included studies, following the Cochrane Handbook for Systematic Reviews of Interventions [25]. Table 3 summarizes the key differences in the survey for a stratified analysis of the risk of heterogeneity assessment of the included studies in this systematic review and meta-analysis. Crocin doses ranged from 5 to 100 mg/d, with most studies using 15–30 mg/d for 6–12 weeks on average, suggesting that these are the most commonly used daily doses. Interventions were applied to adults and the elderly, including those with smoking habits, diabetics with and without complications, individuals under methadone treatment protocols, patients diagnosed with metabolic syndrome, those with coronary diseases, and those living with schizophrenia. This indicates high variability in clinical settings undergoing crocin treatment strategies. Treatment frequencies varied from once to twice daily (demonstrating flexibility in treatment frequency without a fixed standard), and the crocin intervention was compared with a placebo, as well as the health statuses of the included patients, suggesting that interventions with crocin might be of broad interest due to its potential wide-ranging therapeutic applications.

One limitation of our meta-analysis is that the majority of the included studies were conducted in Iran. This concentration of studies in a single country may reduce the ethnic and genetic diversity of the data, limiting the generalizability of our findings to populations from other regions. While we made efforts to include studies from various databases, the majority of the randomized controlled trials were from Iran. We acknowledge that additional studies from different ethnic and geographic populations are needed to assess whether the observed effects of crocin on lipid profiles are consistent across diverse genetic backgrounds. To overcome this limitation, the included patients had diverse conditions, including smoking habits, diabetes (including those with diabetic maculopathy), methadone maintenance treatment, metabolic syndrome, coronary artery disease, and schizophrenia. Future research should aim to include a broader array of ethnic and genetic groups to improve the generalizability of the findings.

### 3.3. Results from the Quantitative Assessments

***Low-Density Lipoprotein Cholesterol (LDL-C)***

**Figure 4:**
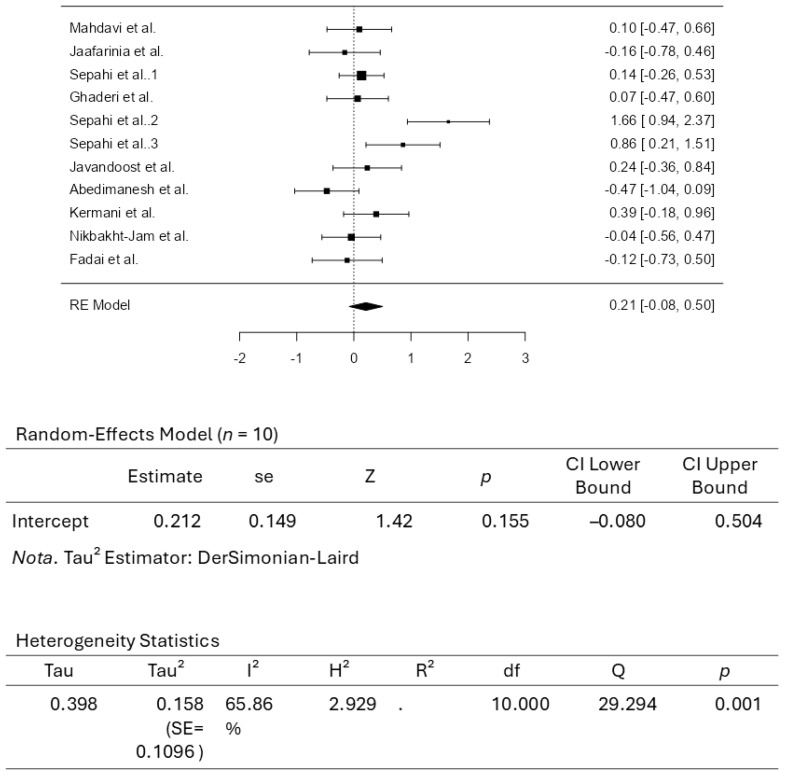
Forest Plot Depicting Crocin Intervention on LDL-C [35,36,37,38,39,40,41,42,43,44].

A total of ten studies comprising eleven results were included in the analysis. The observed standardized mean differences ranged from −0.4735 to 1.6557, with most estimates being positive (64%). The estimated average standardized mean difference based on the random-effects model was 0.2120 (95% CI: −0.0799 to 0.5040). Therefore, the average outcome did not differ significantly from zero (z = 1.4233, *p* = 0.1546). According to the Q-test, the true outcomes appear to be heterogeneous (Q(10) = 29.2945, *p* = 0.0011, tau^2^ = 0.1580, I^2^ = 65.8639%). A 95% prediction interval for the true outcomes is given by −0.6200 to 1.0441. Hence, although the average outcome is estimated to be positive, some studies may yield a negative outcome. An examination of the studentized residuals revealed that the survey conducted by Sepahi et al. [37], which assessed crocin supplementation at 5 mg/d, had a value larger than ± 2.8376 and may be a potential outlier in the context of this model. According to Cook’s distances, the same study by Sepahi et al. (5 mg/d) [37] could be overly influential. Neither the rank correlation nor the regression test indicated any funnel plot asymmetry (*p* = 0.2830 and *p* = 0.0702, respectively). Figure 4 illustrates the forest plot for the meta-analysis, which reports the effects of daily crocin supplementation on LDL-C.

***High-Density Lipoprotein Cholesterol (HDL-C)***

**Figure 5:**
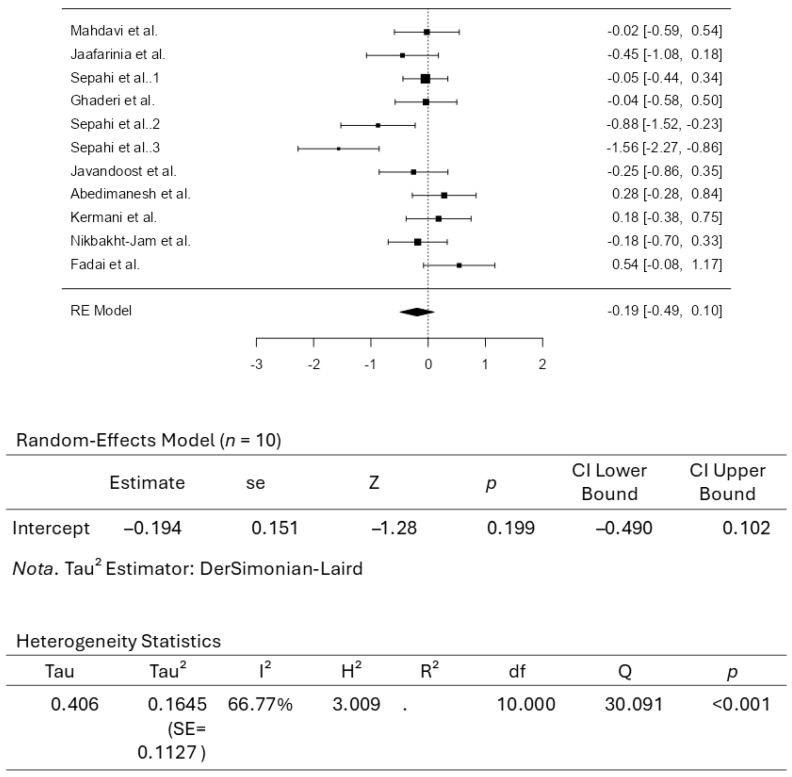
Forest Plot Depicting Crocin Intervention on HDL-C [35,36,37,38,39,40,41,42,43,44].

A total of ten studies comprising eleven results were included in the analysis. The observed standardized mean differences ranged from −1.5639 to 0.5427, with most estimates being negative (73%). The estimated average standardized mean difference based on the random-effects model was −0.1937 (95% CI: −0.4896 to 0.1022). Therefore, the average outcome did not differ significantly from zero (z = −1.2832, *p* = 0.1994). According to the Q-test, the true outcomes appear to be heterogeneous (Q(10) = 30.0913, *p* = 0.0008, tau^2^ = 0.1645, I^2^ = 66.7678%). A 95% prediction interval for the true outcomes is given by −1.0420 to 0.6546. Hence, although the average outcome is estimated to be negative, some studies may yield a positive outcome. An examination of the studentized residuals revealed that the survey conducted by Sepahi et al. [37], which assessed crocin at 15 mg/d, had a value larger than ± 2.8376 and may be a potential outlier in the context of this model. According to the Cook’s distances, none of the studies can be considered overly influential. Neither the rank correlation nor the regression test indicated any funnel plot asymmetry (*p* = 0.1210 and *p* = 0.0747, respectively). Figure 5 illustrates the forest plot for the meta-analysis, which reports the effects of daily crocin supplementation on HDL-C.

***Triglycerides***

**Figure 6:**
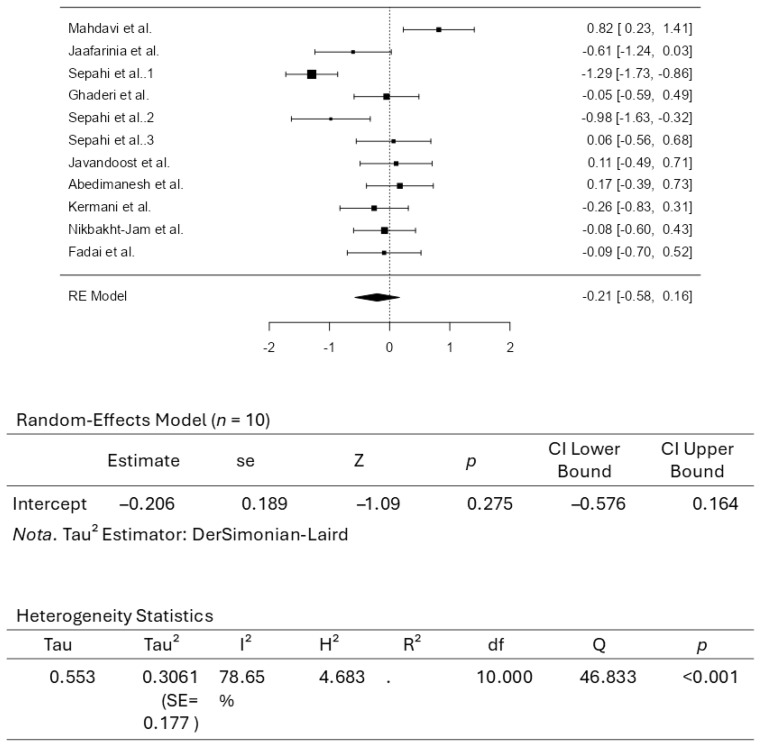
Forest Plot Depicting Crocin Intervention on Triglycerides [35,36,37,38,39,40,41,42,43,44].

A total of ten studies comprising eleven results were included in the analysis. The observed standardized mean differences ranged from −1.2947 to 0.8172, with most estimates being negative (64%). The estimated average standardized mean difference based on the random-effects model was −0.2063 (95% CI: −0.5764 to 0.1638). Therefore, the average outcome did not differ significantly from zero (z = −1.0926, *p* = 0.2746). According to the Q-test, the true outcomes appear to be heterogeneous (Q(10) = 46.8330, *p* < 0.0001, tau^2^ = 0.3061, I^2^ = 78.6476%). A 95% prediction interval for the true outcomes is given by −1.3522 to 0.9395. Hence, although the average outcome is estimated to be negative, some studies may yield a positive outcome. An examination of the studentized residuals revealed that the survey conducted by Sepahi et al. [37], which assessed crocin at 30 mg/d, had a value larger than ± 2.8376 and may be a potential outlier in the context of this model. According to the Cook’s distances, none of the studies can be considered overly influential. Neither the rank correlation nor the regression test indicated any funnel plot asymmetry (*p* = 0.7612 and *p* = 0.3588, respectively). Figure 6 illustrates the forest plot for the meta-analysis, which reports the effects of daily crocin supplementation on triglycerides.

***Total Cholesterol***

**Figure 7:**
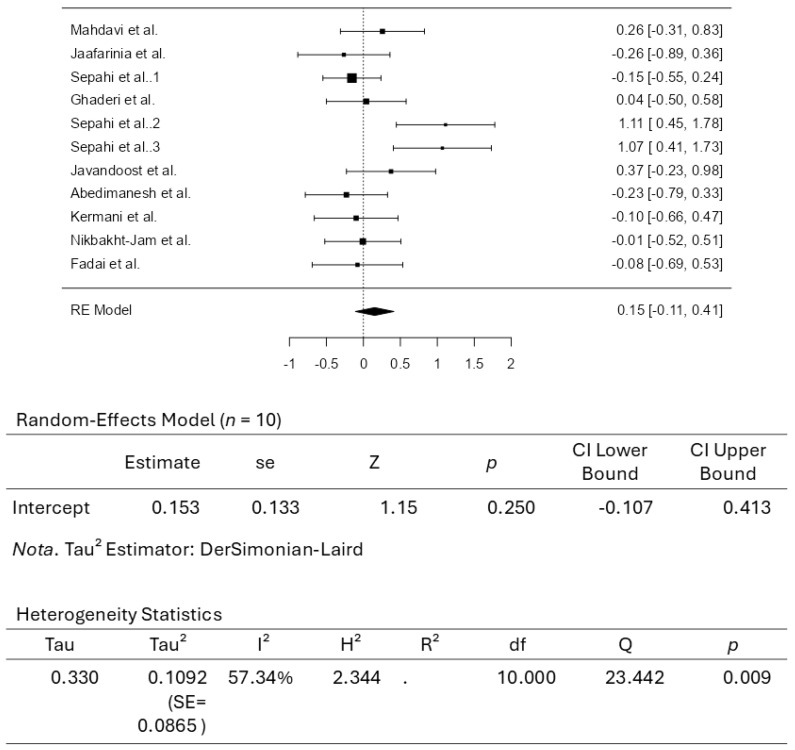
Forest Plot Depicting Crocin Intervention on Total Cholesterol [35,36,37,38,39,40,41,42,43,44].

A total of ten studies comprising eleven results were included in the analysis. The observed standardized mean differences ranged from −0.2639 to 1.1116, with most estimates being negative (55%). The estimated average standardized mean difference based on the random-effects model was 0.1528 (95% CI: −0.1074 to 0.4129). Therefore, the average outcome did not differ significantly from zero (z = 1.1510, *p* = 0.2497). According to the Q-test, the true outcomes appear to be heterogeneous (Q(10) = 23.4416, *p* = 0.0092, tau^2^ = 0.1092, I^2^ = 57.3408%). A 95% prediction interval for the true outcomes is given by −0.5452 to 0.8507. Hence, although the average outcome is estimated to be positive, some studies may yield a negative result. An examination of the studentized residuals revealed that none of the studies had a value larger than ±2.8376, indicating no outliers in the context of this model. According to the Cook’s distances, none of the studies can be considered overly influential. Both the rank correlation and the regression test indicated potential funnel plot asymmetry (*p* = 0.0405 and *p* = 0.0285, respectively). Figure 7 illustrates the forest plot for the meta-analysis, which reports the effects of daily crocin supplementation on total cholesterol.

***Dose Response Assessment***

**Figure 8:**
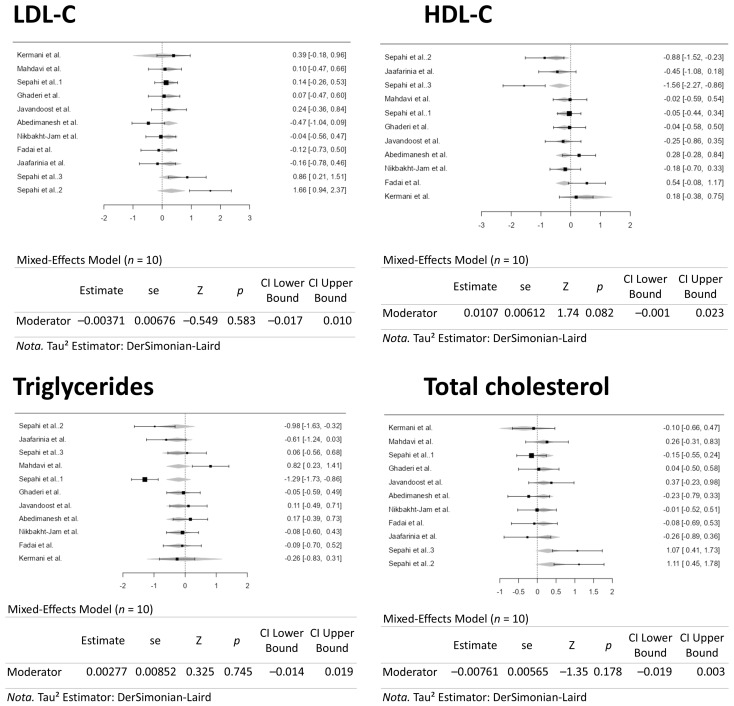
Forest Plots Depicting the Effect of Crocin Intervention on Blood Lipids Following a Dose–Response [35,36,37,38,39,40,41,42,43,44].

A dose–response meta-analysis was conducted to assess the impacts of crocin (ranging from 5 to 100 mg/d) on the outcomes related to HDL-C, triglycerides, LDL-C, and total cholesterol in the current study. Figure 8 presents the forest plots resulting from these analyses. The regulation of crocin supplementation dosages did not influence the outcome measures. Consequently, the consistency of the supplement dosage did not alter the previously reported results.

***Period of Intervention (Time)–Response Assessment***

**Figure 9:**
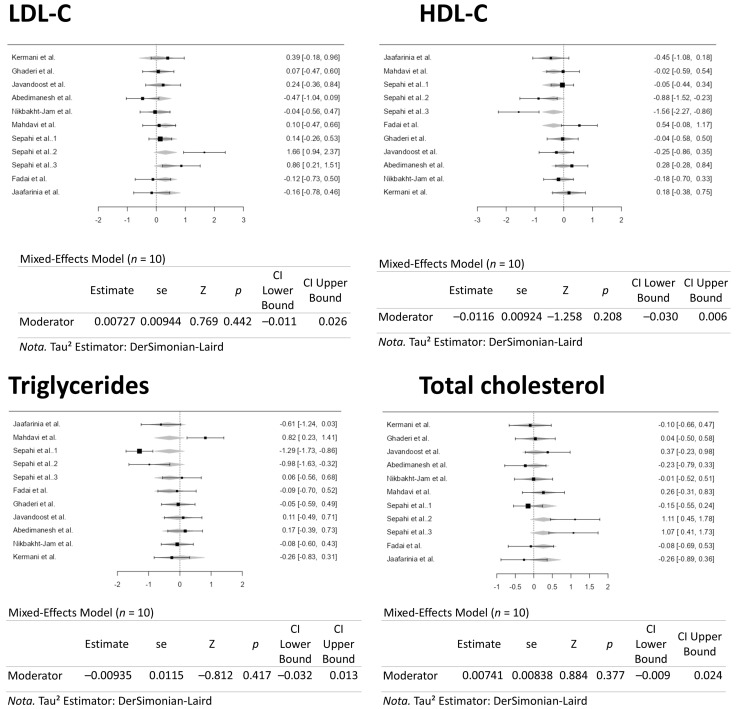
Forest Plots Depicting the Effect of Crocin Intervention on Blood Lipids over Time Following a Time–Response [35,36,37,38,39,40,41,42,43,44].

A time–response meta-analysis was conducted to assess the impacts of crocin (ranging from 5 to 100 mg/d) on the outcomes related to HDL-C, triglycerides, LDL-C, and total cholesterol in the current study. Figure 9 presents the forest plots resulting from these analyses. The regulation of crocin supplementation periods did not influence the outcome measures. Consequently, the consistency of the supplement period did not alter the previously reported results.

## 4. Conclusions

**Figure 10:**
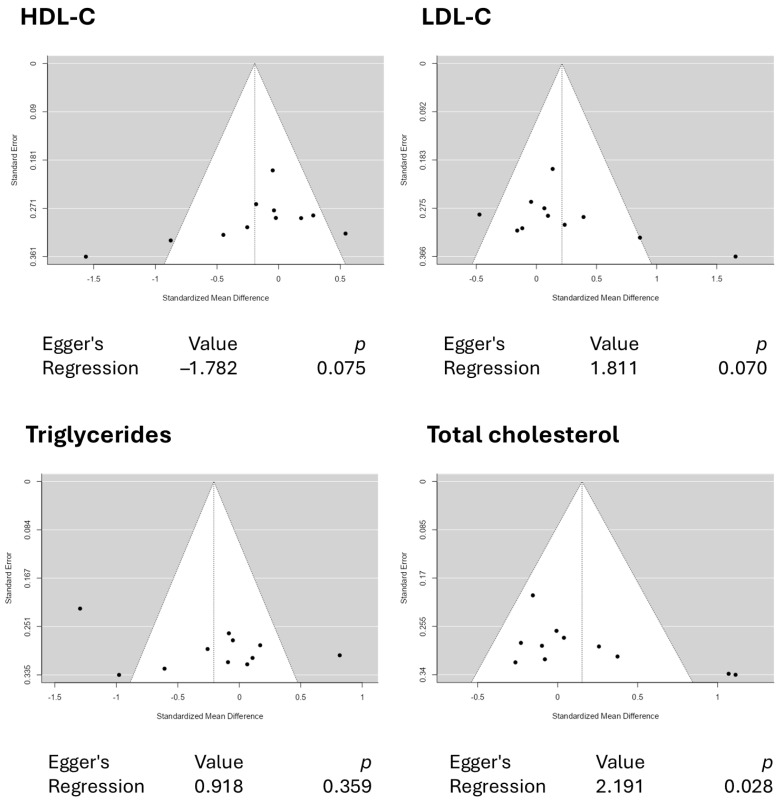
Funnel plots depicting no asymmetry from crocin interventions on HDL-C, LDL-C, and triglyceride levels. The funnel plot revealed asymmetry for total cholesterol.

This systematic review and meta-analysis aimed to evaluate the efficacy of crocin in modulating blood lipid levels and to explore potential dose- and time-dependent effects. Although crocin is widely recognized for its antioxidant and anti-inflammatory properties, which mechanistically support lipid-lowering effects, our analysis of ten studies and eleven outcomes’ datasets revealed no statistically significant improvements in LDL-C, HDL-C, total cholesterol, and triglyceride levels. Likewise, neither the dose–response nor the time–response trends reached significance. These findings highlight a discrepancy between the proposed mechanisms of action of crocin, its potential benefits, and its real clinical outcomes. The current body of evidence remains limited by relatively small sample sizes, variability in study quality, short intervention periods, and heterogeneity in dosage regimens and participant populations. This underscores the necessity of conducting more rigorous and well-designed randomized clinical trials with diverse populations and standardized dosing protocols, more extended follow-up periods, and adequate statistical power to definitively assess crocin’s impact on lipid metabolism. Besides the non-significant findings, our meta-analysis revealed funnel plot asymmetry only for total cholesterol, but not for HDL-C, LDL-C, or triglycerides. Figure 10 illustrates the funnel plots for the meta-analysis, which revealed asymmetry for total cholesterol but not for HDL-C, LDL-C, or triglycerides, highlighting the reliability of the quantitative analyses for these latter outcomes.

Future research endeavors should consider stratifying participants based on baseline lipid profiles, comorbidities, and genetic polymorphisms that may influence lipid metabolism or antioxidant responsiveness. Additionally, exploring crocin’s synergistic potential with other bioactive compounds or pharmacotherapies may reveal combinatory effects not observable in isolation. Conducting large-scale, multi-center randomized controlled trials with standardized dosing regimens would undoubtedly overcome current limitations in sample size, study quality, and heterogeneity, thereby increasing the reliability and generalizability of results across diverse populations. Investigating crocin’s effectiveness in reducing blood lipids across diverse specific subpopulations, for example, in individuals suffering from metabolic syndrome, diabetes, or genetic lipid disorders, might also be essential to detect more pronounced effects of crocin as a lipid-lowering agent in individuals with differing metabolic dysfunctions, especially where inflammation and oxidative stress are more pronounced. Investigating crocin’s mechanisms of action in human subjects is also of utmost importance. This would clarify crocin’s bioavailability, metabolism, and direct molecular targets in lipid regulation, thereby bridging the gap between proposed mechanisms of action and observed clinical effects. Finally, researchers must conduct long-term safety and tolerability studies of crocin supplementation in clinical populations to ensure that chronic use of crocin is safe and free of adverse effects, which is critical for its consideration as long-term therapy for dyslipidemia. In this scenario, dose– and time–response designs would be beneficial in enhancing the statistical power to refine the understanding of crocin’s efficacy thresholds and determine optimal dosages and minimum effective durations, which can inform clinical guidelines and improve intervention outcomes.

In addition to the results above, it is essential to highlight some critical advancements in dyslipidemia research that have garnered significant attention. Traditionally, high levels of HDL-C are regarded as beneficial [45]. This is due to the particle’s role in reversing cholesterol transport, which helps remove excess cholesterol from tissues and reduces the risk of cardiovascular disease [46,47]. However, recent studies suggest that excessively high HDL-C levels, particularly those above 116 mg/dL in women and 94 mg/dL in men, may not provide additional protective effects and could even be associated with adverse health outcomes. Some research has shown that very high HDL-C levels might reflect dysfunction in HDL-C particles, which can become pro-inflammatory and pro-atherogenic [48,49,50,51,52]. These findings challenge the notion that higher HDL-C levels are always beneficial, suggesting that the functionality of HDL-C particles may be just as necessary as their concentration in the blood [53,54]. Therefore, understanding crocin’s roles in influencing HDL-C functionality, rather than its blood levels, in improving lipid metabolism and cardiovascular health is also necessary.

The functionality and protective properties of HDL-C are influenced by several factors, including its lipid and protein composition, particle size, and modifications such as oxidation and glycation [55]. For example, HDL-C particles with a higher amount of specific proteins, such as apoA-I, tend to exhibit superior anti-inflammatory and antioxidant properties, which contribute to their protective role against cardiovascular diseases. Conversely, oxidized and glycated HDL-C particles have been shown to exacerbate cellular senescence, contributing to the pathogenesis of atherosclerosis. A healthier HDL-C is enriched with cholesterol and apoA-I, characterized by a distinct round shape and a larger particle size. Conversely, the worst HDL-C particle exhibits apoA-I displacement, accompanied by cholesterol decrease and enrichment of triglycerides, human serum amyloid A, and apoC-III [52].

Despite the growing body of evidence supporting the cardiovascular benefits of crocin on lipid parameters, its effects on lipoprotein a, Lp(a), are still not thoroughly examined. Lp(a) acts by inducing vascular inflammation, atherogenesis, calcification, and thrombus formation [56,57]. Elevated plasma levels of Lp(a) are an independent and causal risk factor for atherosclerosis and atherosclerosis-related cardiovascular diseases and outcomes. Lp(a) resembles LDL-C, and novel therapeutics have been proposed to specifically lower Lp(a) serum levels [58,59]. Since crocin presents lipid-lowering effects, studying this compound against higher Lp(a) levels may be of utmost importance for the advancement of this field of science.

In summary, while crocin remains a promising candidate within the landscape of nutraceuticals for dyslipidemia management, its clinical efficacy is yet to be conclusively demonstrated. High-quality evidence is essential before recommending it as a routine lipid-modulating agent. Our meta-analysis makes several significant contributions to the field. Although we found no significant results regarding overall lipid markers, our study is the first to provide a comprehensive dose–response analysis and investigate potential moderators of treatment effects, such as dosage and duration. Additionally, by combining mechanistic data with clinical outcomes, we present a unique perspective on the effects of crocin. These findings suggest that while crocin shows promise, more rigorously controlled studies are necessary to evaluate its therapeutic potential fully.

## Figures and Tables

**Table 1:** Report of the Included Studies Following PRISMA Guidelines.

Study	Country	Study Design	Participants	Sex (M/F)	Sample Size		Trial Duration	Age (Years)		BMI (kg/m^2^)		Intervention		
					IG	CG		IG	CG	IG	CG	Type	Dose (mg/d)	Control Group
Mahdavi et al. [35]	Iran	RCT, double-blind	Smokers	39 M/9 F	23	25	3 months	34.39 ± 10.72	36.20 ± 9.23	NR	NR	Tablets	30	Placebo
Jaafarinia et al. [36]	Iran	RCT, triple-blind	T2DM	23 M/17 F	21	19	90 days	63.86 ± 10.62	62.68 ± 9.84	27.21 ± 3.86	27.26 ± 3.34	Tablets	15	Placebo
Sepahi et al. [37]	Iran	RCT, triple-blind	T2DM	47 M/53 F	50	50	3 months	57.58 ± 1.0	56.92 ± 1.9	NR	NR	Tablets	30	Placebo
Ghaderi et al. [38]	Iran	RCT, double-blind	MMT	NR	26	27	8 weeks	44.5 ± 9.4	45.6 ± 9.9	24.5 ± 4.4	25.2 ± 4.2	Tablets	30	Placebo
Sepahi et al. [39]	Iran	RCT, double-blind	Diabetic maculopathy	29 M/31 F	40	20	3 months	54.31 ± 6.6 (5 mg/d), 56.09 ± 4.3 (15 mg/d)	57.17 ± 2.9	NR	NR	Tablets	5 or 15	Placebo
Kermani et al. [40]	Iran	RCT, double-blind	MetS	7 M/41 F	24	24	6 weeks	53.8 ± 9.2	50.9 ± 8.8	29.9 ± 3.9	29.8 ± 5.3	Tablets	100	Placebo
Javandoost et al. [41]	Iran	RCT, double-blind	MetS	18 M/26 F	21	22	8 weeks	33.10 (M) and 44.5 (F)	34.9 (M) and 46.0 (F)	NR	NR	Tablets	30	Placebo
Abedimanesh et al. [42]	Iran	RCT, double-blind	CAD	25 M/25 F	25	25	8 weeks	53.36 ± 5.94	56.32 ± 5.91	27.92 ± 2.57	28.05 ± 2.89	Capsules	30	Placebo
Nikbakht-Jam et al. [43]	Iran	RCT, double-blind	MetS	25 M/33 F	29	29	8 weeks	38.97 ± 13.33	43.46 ± 12.77	NR	NR	Tablets	30	Placebo
Fadai et al. [44]	Iran	RCT, triple-blind	Schizophrenia	41 M	20	21	12 weeks	48.1 ± 7.7	48.1 ± 6.1	NR	NR	Capsules	30	Placebo

**Abbreviations:** BMI, Body mass index; CAD, Coronary artery disease; CG, Control group; F, Female (women); IG, Interventional group; M, Male (men); MMT, Methadone maintenance treatment; MetS, Metabolic syndrome; NR, Not reported; RCT, Randomized controlled trial; T2DM, Type 2 diabetes mellitus.

**Table 2:** Report of bias identification throughout the studies following the Cochrane Handbook for Intervention Assessment.

Study	Question Focus	Appropriate Randomization	Allocation Blinding	Double-Blind	Losses (<20%)	Prognostic or Demographic Characteristics	Outcomes	Intention-to-Treat Analysis	Sample Calculation	Adequate Follow-Up
Mahdavi et al. [35]	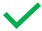	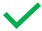	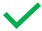	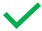	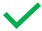	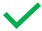	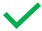	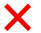	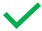	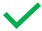
Jaafarinia et al. [36]	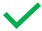	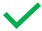	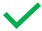	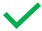	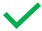	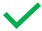	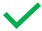	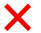	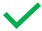	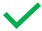
Sepahi et al. [37]	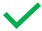	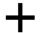	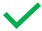	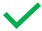	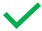	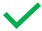	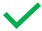	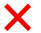	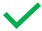	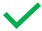
Ghaderi et al. [38]	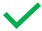	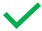	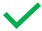	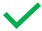	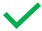	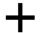	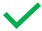	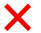	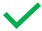	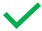
Sepahi et al. [39]	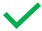	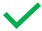	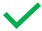	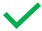	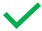	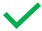	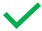	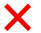	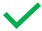	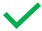
Kermani et al. [40]	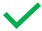	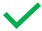	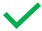	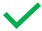	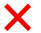	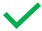	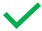	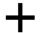	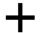	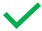
Javandoost et al. [41]	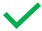	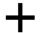	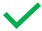	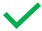	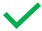	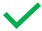	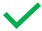	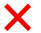	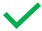	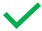
Abedimanesh et al. [42]	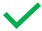	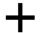	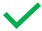	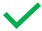	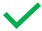	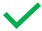	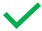	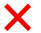	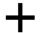	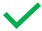
Nikbakht-Jam et al. [43]	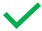	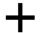	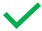	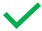	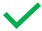	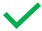	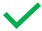	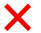	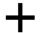	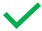
Fadai et al. [44]	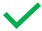	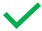	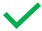	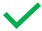	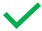	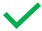	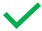	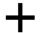	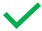	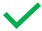

**Abbreviations:** 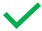, Low risk of bias; 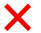, High risk of bias; 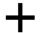, Unclear risk of bias.

**Table 3:** Summary of key study differences for stratified analysis.

Stratification Category	Observed Variations Across the Included Studies
Crocin dosage	Ranged from 5–100 mg daily, with the majority of the studies applying doses from 15–30 mg/d.
Intervention duration	Ranged principally from 6 to 12 weeks.
Population age	Ranged from adults (~33.10 years) to elderly (63.86 ± 10.62 years) individuals.
Health status	The intervention protocols included smokers, diabetic individuals (including those with diabetic complications like diabetic maculopathy), individuals under methadone maintenance treatment, individuals with metabolic syndrome, patients with coronary artery disease, and individuals living with schizophrenia.
Intervention protocols	Varied principally regarding frequency of the interventions (once vs. twice daily) and administration timing.
Subgroup analysis performed	Conducted mainly regarding crocin dosage or treatment vs. placebo and health statuses of the included participants.

## Data Availability

The datasets responsible for the results from this updated systematic review and meta-analysis will be made available for readers upon request to the corresponding author.
